# Predicting clustered weather patterns: A test case for applications of convolutional neural networks to spatio-temporal climate data

**DOI:** 10.1038/s41598-020-57897-9

**Published:** 2020-01-28

**Authors:** Ashesh Chattopadhyay, Pedram Hassanzadeh, Saba Pasha

**Affiliations:** 1 Rice University, Houston, 77005 USA; 2 University of Pennsylvania, Philadelphia, 19104 USA

**Keywords:** Atmospheric dynamics, Climate and Earth system modelling, Projection and prediction, Environmental impact

## Abstract

Deep learning techniques such as convolutional neural networks (CNNs) can potentially provide powerful tools for classifying, identifying, and predicting patterns in climate and environmental data. However, because of the inherent complexities of such data, which are often spatio-temporal, chaotic, and non-stationary, the CNN algorithms must be designed/evaluated for each specific dataset and application. Yet CNN, being a supervised technique, requires a large labeled dataset to start. Labeling demands (human) expert time which, combined with the limited number of relevant examples in this area, can discourage using CNNs for new problems. To address these challenges, here we (1) Propose an effective auto-labeling strategy based on using an unsupervised clustering algorithm and evaluating the performance of CNNs in re-identifying and predicting these clusters up to 5 days ahead of time; (2) Use this approach to label thousands of daily large-scale weather patterns over North America in the outputs of a fully-coupled climate model and show the capabilities of CNNs in re-identifying and predicting the 4 clustered regimes up to 5 days ahead of time. The deep CNN trained with 1000 samples or more per cluster has an accuracy of 90% or better for both identification and prediction while prediction accuracy scales weakly with the number of lead days. Accuracy scales monotonically but nonlinearly with the size of the training set, e.g. reaching 94% with 3000 training samples per cluster for identification and 93–76% for prediction at lead day 1–5, outperforming logistic regression, a simpler machine learning algorithm, by  ~ 25%. Effects of architecture and hyperparameters on the performance of CNNs are examined and discussed.

## Introduction

Classifying, identifying, and predicting specific patterns or key features in spatio-temporal climate and environmental data are of great interest for various purposes such as finding circulation regimes and teleconnection patterns^1–5^, identifying extreme-causing weather patterns^6–12^, studying the effects of climate change^13–16^, understanding ocean-atmosphere interaction^8,17,18^, weather forecasting^8,12,19,20^, and investigating air pollution transport^21,22^, just to name a few. Such classifications/identifications and predictions are often performed by employing empirical orthogonal function (EOF) analysis, clustering algorithms (e.g., K-means, hierarchical, self-organizing maps^1,3,23–29^), linear regression, or specifically designed indices, such as those used to identify atmospheric blocking events. Each approach suffers from some major shortcomings (see the reviews by Grotjahn *et al*.^6^ and Monahan *et al*.^30^); for example, there are dozens of blocking indices which frequently disagree and produce conflicting statistics on how these high-impact extreme-causing weather patterns will change with climate change^10,14,31^.

In recent years, applications of machine learning methods for accelerating and facilitating scientific discovery have increased rapidly in various research areas. For example, in climate science, neural networks have produced promising results for parameterization of convection and simulation of clouds^32–36^, and forecasting of weather/climate variability and extremes and weather forecasting^12,20,37–42^; also see the recent Perspective by Reichstein *et al*.^43^. A class of supervised deep learning architectures, called convolutional neural networks (CNN), has transformed pattern recognition and image processing in various domains of business and science^44,45^ and can potentially become a powerful tool for classifying and identifying patterns in climate and environmental data^43^. In fact, in their pioneering work, Liu *et al*.^46^ and Racah *et al*.^47^ have shown the promising capabilities of CNNs in identifying tropical cyclones, weather fronts, and atmospheric rivers in large, labeled climate datasets.

Despite the success in applying CNNs in these few studies, there are some challenges that should be addressed to further expand the applications and usefulness of CNNs (and similar deep learning techniques) in climate and environmental sciences^48^. One major challenge is that unlike the data traditionally used to develop and assess CNN algorithms such as the static images in ImageNet^49^, climate and environmental data, from model simulations or observations are often spatio-temporal, highly nonlinear, chaotic, high-dimensional, non-stationary, multi-scale, and correlated. For example, the large-scale atmospheric circulation, whose variability strongly affects day-to-day weather and extreme events, is a high-dimensional turbulent system with length scales of smaller than 1 m to larger than 10000 km and time scales of minutes to decades (and beyond), with strongly coherent and correlated patterns due to various physical processes, and non-stationarity due to, e.g., atmosphere-ocean coupling and anthropogenic effects^50–52^. An additional challenge with observational datasets is that they are usually short and sparse and have measurement noise.

As a result, to fully harness the power of CNNs (or similar deep learning techniques), the algorithms (architecture, hyperparameters etc.) have to be designed and evaluated for each specific climate or environmental data and for each specific application. However, to start, CNN, as a supervised technique, requires a large labeled dataset for training/testing. Labeling data demands (human) expert time and resources and while some labeled datasets for specific types of data and applications are now publicly available^47,53^, it can discourage exploring the capabilities of CNN for various problems. With these challenges in mind, the purpose of this paper is two-fold:

1. To propose an effective, simple, and algorithmic approach for labeling any spatio-temporal climate and environmental data based on using an easy-to-implement unsupervised clustering technique. The large, labeled dataset accelerates the exploration and application of CNNs (and similar methods) to complex research problems in climate and environmental sciences,
2. To use this approach in a test case and label thousands of large-scale weather patterns over North America in the outputs of a state-of-the-art climate model, show the capabilities of CNNs in re-identifying the clustered patterns and predicting their time evolution, and examine how the performance of CNNs depend on the architecture, hyperparameters, and size of the training dataset.

## Methodology

The approach proposed here involves two steps: (i) the spatio-temporal data is clustered into *n* classes using an unsupervised technique such as K-means^54^, which assigns an index (1 to *n*) to each pattern in the dataset, and (ii) the cluster indices are used to label the patterns in the dataset, 1 to *n* for day 0, day  − 1, ...  day  − 5 and so on. The labeled dataset is then used to train and test the CNN. The performance of CNN in re-identifying (for day 0) which cluster index a pattern belongs to, or predicting which cluster index a given pattern will evolve to in a few days, can be used to evaluate and explore improvements to the CNN algorithms for each specific dataset. Note that here we use K-means clustering for indexing, but other algorithms such as hierarchical, expectation-maximization, or self-organizing maps^3,4,23–29^ can be used instead. However, the K-means algorithm, which clusters the data into *a priori* specified *n* classes based on Euclidean distances, provides an effective, simple method for the objective here, which is labeling the dataset for evaluating CNN, as opposed to finding the most meaningful (if even possible^17^) number of clusters in the spatio-temporal data.

The approach proposed here can be used for any climate or environmental data such as wind, precipitation, or sea-surface temperature patterns or distributions of pollutants, to name a few. For the case study presented here, we focus on re-identifying and predicting the daily weather patterns over North America in summer and winter. The data, the K-means clustering and CNN algorithms are presented in Data and Methods, but we discuss their key aspects briefly below. We use data from the Large Ensemble (LENS) Community Project^55^, which consists of a 40-member ensemble of fully-coupled Community Earth System Model version 1 (CESM1) simulations with historical radiative forcing from 1920 to 2005. We focus on daily averaged geopotential height at 500 hPa (Z500 hereafter), whose isolines are approximately the streamlines of the large-scale circulation at mid-troposphere and are often used to represent weather patterns^56^. Daily Z500 from 1980 to 2005 provides  ~95000 samples for summer months and for winter months over North America. The advantage of using this large-ensemble dataset is that the simulated patterns have complexity similar to those of the real atmosphere, while a large number of samples are available for evaluating the CNN architectures, and in particular, the scaling of the accuracy with the size of the training set.

**Figure 1:**
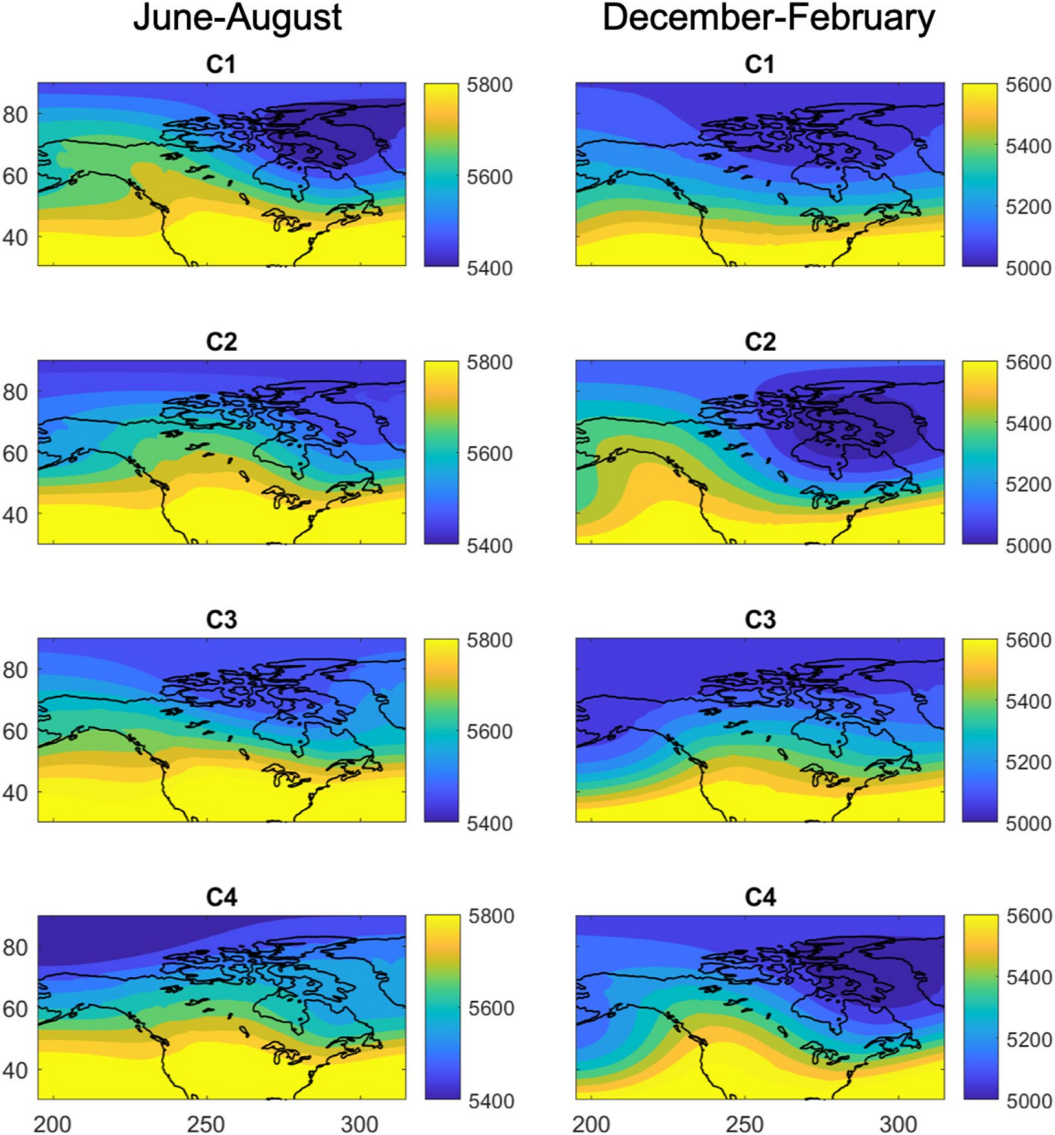
Centers of the four K-means clusters in terms of the full Z500 field (with unit of meters) at day 0 for summer months, June-August (left column) and for winter months, December-February (right column). The K-means algorithm finds the cluster centers based on *a priori* specified number of clusters *n* (=4 here) and assigns each daily pattern to the closest cluster center based on Euclidean distances for that day (day 0). The assigned cluster indices are used as labels for training/testing CNNs (a different CNN for each day). Note that K-means clustering is performed on daily zonal-mean-removed Z500 anomalies projected onto their first 22 EOFs, but the cluster indices are used to label the full Z500 patterns to minimize pre-processing and retain the complex temporal variabilities of the Z500 field (see Data and Methods for further discussions).

As discussed in Data and Methods, the K-means algorithm is used to classify the winter days and summer days (separately) into *n* = 4 clusters. The clustering analysis is performed on zonal-mean-removed daily Z500 anomalies projected on 22 EOFS that retain approximately 95% of the variance; however, the computed cluster index for each day is used to label the full Z500 pattern of that day and 5 days earlier. The four cluster centers in terms of the full Z500 field for day 0 are shown in Fig. 1. Labeled full Z500 patterns are used as input to CNN for training and testing for day 0, day  − 1 ⋯  day  − 5. We work with the full Z500 fields, rather than the computed anomalies (or any other type of anomalies), because one hopes to use CNN with minimally pre-processed data. Therefore, we focus on the more difficult task of re-identifying and predicting the clusters in the full Z500 fields, which include complex temporal variabilities such as the seasonal cycle and non-stationarity resulting from the low-frequency coupled ocean-atmosphere modes and changes in the radiative forcing between 1980 and 2005. We further emphasize that the spatio-temporal evolution of Z500 field is governed by high-dimensional, strongly nonlinear, chaotic, multi-scale dynamics^56^.

**Figure 2:**
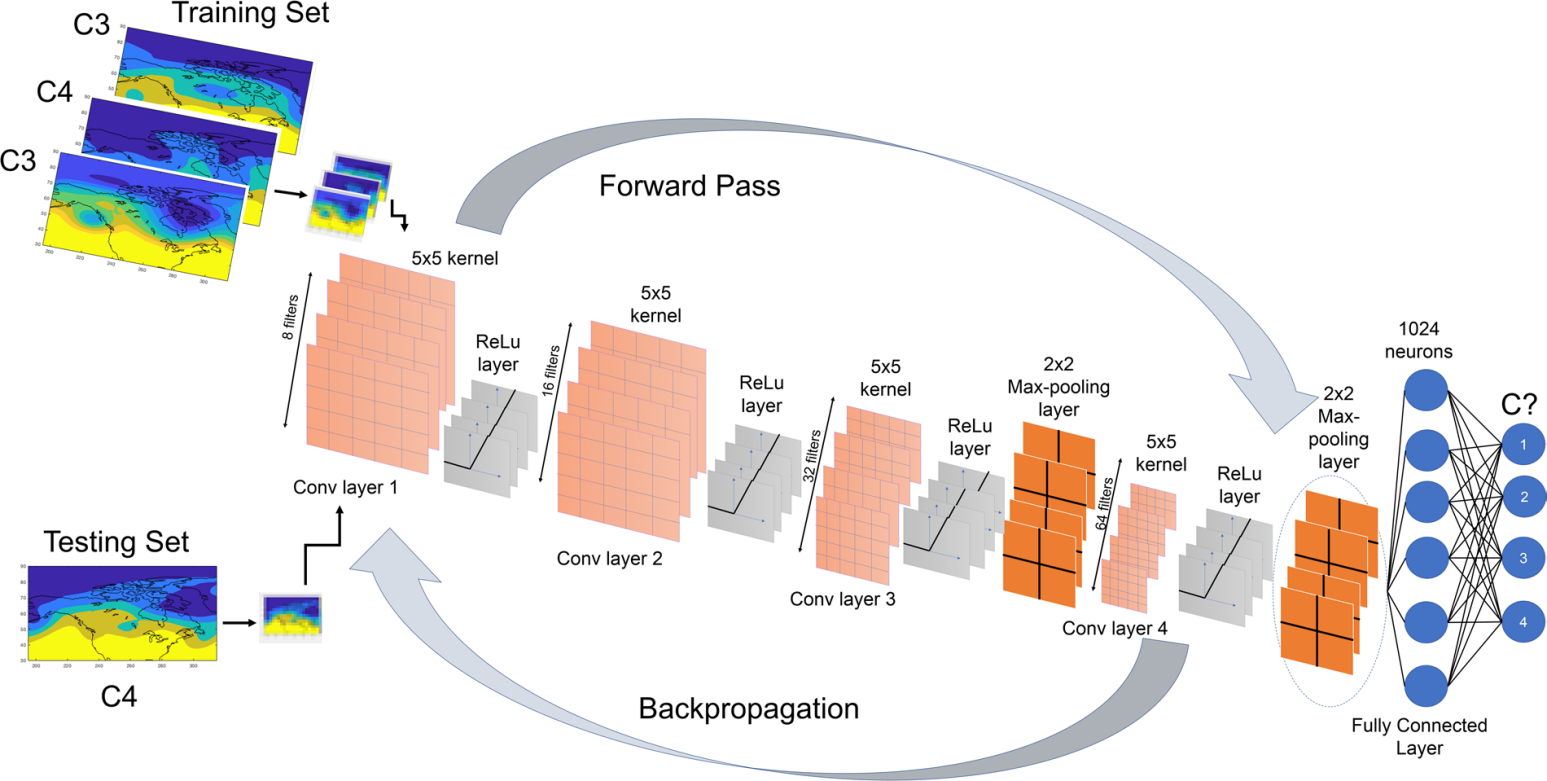
The architecture of CNN4, which has 4 convolutional layers that have 8,16,32 and 64 filters, respectively. Each filter has a kernel size of 5 × 5. Filters of the max-pooling layer have a kernel size of 2 × 2. The convolutional layers at the beginning capture the low-level features while the latter layers would pick up the high level features^77^. Each convolution step is followed by the ReLU layer that introduces nonlinearity in the extracted features. In the last two layers, a max-pooling layer after the ReLU layer retains only the most dominant features in the extracted feature map while inducing translational and scale invariance. These extracted feature maps are then concatenated into a single vector which is connected to a fully connected neural network with 1024 neuron. The output is the probability of each class. The input images into this network have been first down-sampled using bi-cubic interpolation to only retain the large-scale features in the circulation patterns (Fig. 3).

**Figure 3:**
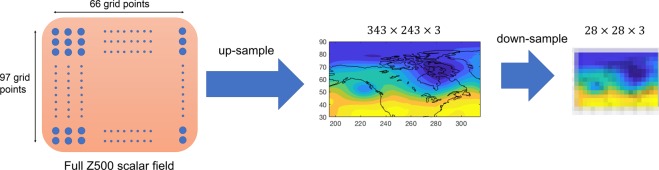
Schematic of the up-sampling and down-sampling steps. Each daily full Z500 pattern, which is on a 66 × 97 latitude-longitude grid, is converted to a contour plot represented by a RGB image of size 342 × 243 pixels with 3 channels representing red, green, and blue. This up-sampled image is then down-sampled to an image of size 28 × 28 × 3 using bi-cubic interpolation and further standardized by subtracting the mean and dividing by the standard deviation of the pixel intensities. These images are the inputs to CNN for training or testing. The down-sampling step is used to remove redundant features at small scales from each sample. Trying to learn such small features, which are mostly random, can result in overfitting of the network. Note that rather than converting the data matrix into a RGB image, CNN could be applied directly to the data matrix, which we have found to yield the same accuracies. See Data and Methods for further discussions.

The architecture of our CNN algorithm is shown in Fig. 2. In general, the main components of a CNN algorithm are: convolutional layers in which a specified number of kernels (filters) of specified sizes are applied to extract the key features in the data and produce feature maps; Rectified Linear Unit (ReLU) layers in which the ReLU activation function, $f(x)=\max (0,x)$, is applied to the feature maps to introduce nonlinearity; pooling layers that reduce the dimensions of the feature maps to increase the computational performance, control overfitting, and induce translational and scale invariance (which is highly desirable for the chaotic spatio-temporal data of interest here); and finally, fully connected layers^44,45^. The inputs to CNN are the full Z500 fields that are converted to images and down-sampled to reduce redundancies in small scales (Fig. 3). During the training phase, the images and their cluster indices at day 0 (for re-identification) or the images and the cluster index of a few days later (for prediction), from a randomly drawn training set (TR), are inputted into CNN and the kernels (i.e. their weights) are learned using backpropagation^45^. The major advantage of CNNs over traditional image processing methods is that the appropriate kernels are learned for each dataset, rather than being hand-engineered and specified *a priori*. During the testing phase, images, from a randomly drawn testing set (TS) that has no overlap with TR, are inputted into the CNN and the output is the predicted cluster index. If the CNN has learned the key features of these high-dimensional, nonlinear, chaotic, non-stationary patterns, then the re-identified or predicted cluster indices should be largely correct.

In this paper we developed two CNNs, one with two convolutional layers (CNN2) and another one with four convolutional layers (CNN4). The effects of hyperparameters and other practical issues as well as scaling of the accuracy with the size of the training set are examined and discussed.

## Results

### Performance of CNN for re-identification

Tables 1 and 2 show the test accuracies of CNN2 and CNN4 for the summer and winter months, respectively, for re-identification (day 0). The CNN4 has an accuracy of 93.3% ± 0.2% (summer) and 93.8% ± 0.1% (winter) while CNN2 has an accuracy of 89.0% ± 0.3% (summer) and 86.6% ± 0.3% (winter). The reported accuracies are the mean and standard deviation of the accuracies of the 5 sets in the TS. The 4–7% higher accuracy of the deeper net, CNN4, comes at the price of higher computational demands (time and memory) because of the two additional convolutional layers; however, the robust test accuracy of ~93% is significant for the complex patterns studied here.

**Table 1:** The confusion matrix for CNN4 (CNN2) applied to summer months. A TR of length *N* = 12000 (3000 samples per cluster) and a TS, consisting of 5 independent sets each with 1000 samples per cluster, are used (see Data and Methods). The TR and TS are randomly selected and have no overlap. Each number shows how many patterns from a given cluster in TS are identified by the trained CNN to belong to each cluster (the mean and standard deviation from the 5 sets of TS are reported). The results are from the best trained CNN2 and CNN4. The overall test accuracy, calculated as the sum of the diagonal numbers, i.e. all correctly identified patterns, divided by the total number of patterns, i.e. 3000, and turned to percentage is 93.3% ± 0.2% (CCN4) and 89.0% ± 0.3% (CNN2).

	Identified as C1	Identified as C2	Identified as C3	Identified as C4
True C1	**915** ± **3** (**959** ± **8**)	30 ± 3 (14 ± 4)	55 ± 3 (27 ± 2)	0 ± 0 (0 ± 0)
True C2	17 ± 2 (78 ± 3)	**906** ± **3** (**819** ± **2**)	48 ± 2 (58 ± 2)	29 ± 3(45 ± 4)
True C3	17 ± 1 (61 ± 1)	8 ± 1 (30 ± 1)	**955** ± **3** (**857** ± **3**)	20 ± 2 (52 ± 2)
True C4	0 ± 0 (0 ± 0)	18 ± 2 (49 ± 2)	26 ± 3 (23 ± 2)	**956** ± **3** (**928** ± **3**)

**Table 2:** Same as Table 1 but for winter months. The overall test accuracy is 93.8% ± 0.1% (CNN4) and 86.6% ± 0.3% (CNN2).

	Identified as C1	Identified as C2	Identified as C3	Identified as C4
True C1	**937** ± **2** (**783** ± **3**)	13 (98 ± 1)	37 ± 1 (20 ± 1)	13 ± 1 (99 ± 1)
True C2	71 ± 2 (28 ± 2)	**920** ± **2** (**951** ± **2**)	0 ± 0 (0 ± 0)	9 ± 1 (21 ± 1)
True C3	49 ± 2 (61 ± 1)	0 ± 0 (0 ± 0)	**984** ± **3** (**822** ± **2**)	3 ± 0 (129 ± 0)
True C4	23 ± 0 (4 ± 0)	29 ± 2 (82 ± 2)	37 ± 2 (3 ± 1)	**911** ± **2** (**911** ± **3**)

Deep CNNs are more vulnerable to overfitting: the large number of parameters can lead to perfect accuracy on the training samples while the trained CNN fails to generalize and accurately classify the new unseen samples. In order to ensure that the reported high accuracies of CNNs here are not due to overfitting, during the training phase, a randomly chosen validation set (which does not have any overlap with TS or TR) was used to tune the hyperparameters (see Data and Methods). For each case in Tables 1 and 2, the reported test accuracy is approximately equal to the training accuracy after the network converges which, along with small standard deviations among the 5 independent sets in the TS, indicates that the classes have been learned rather than overfitted. It should be mentioned that for this data with the TR of size *N* ≤ 12000, we have found that overfitting occurs if more than 4 convolutional layers are used.

### Scaling of the test accuracy with the size of the training set

**Figure 4:**
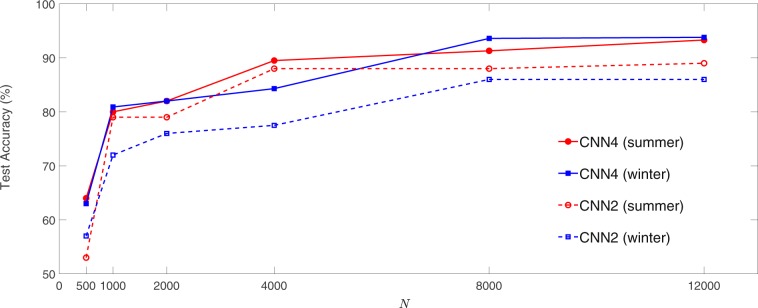
Test accuracy of CNN4 and CNN2 as a function of the size of the training set *N*. To avoid class imbalance, *N*∕4 samples per cluster are used. A 3:1 ratio between the number of samples per cluster in the training and testing sets are maintained.

An important practical question that many ask before investing in labeling data and developing their CNN algorithm is “how much data do I need to get reasonable accuracy with CNN?”. However, a theoretical understanding of the bound or scaling of CNNs’ accuracy based on the number of the training samples or number of tunable parameters of the network is currently unavailable^57^. Given the abundance of the labeled samples in our dataset, it is an interesting experiment to examine how the test accuracy of CNNs scales with the size of the TR, *N*. Figure 4 shows that the test accuracy of CNN2 and CNN4 scales monotonically but nonlinearly with *N* for summer and winter months. With *N* = 500 (125 training samples per cluster), the test accuracy of CNN4 is around 64%. The accuracy jumps above 80% with *N* = 1000 and then increases to above 90% as *N* is increased to 8000. Further increasing *N* to 12000 slightly increases the accuracy to 93%. The accuracy of CNN2 qualitatively shows the same behavior, although it is consistently lower than the accuracy of CNN4 for the same *N*. While the empirical scaling presented here is most likely problem-dependent and cannot replace a theoretical scaling, it provides an example of how the test accuracy might depend on the size of the TR.

The analyses presented so far show how the auto-labeling strategy can be used to accelerate the exploration and application of CNNs (and similar supervised deep learning techniques) to new complex datasets. Next we show the performance of CNN in predicting the evolution of weather patterns and compare it with the performance of another machine learning technique, logistic regression^58^. But before looking at prediction, which is much more challenging than re-identification, we first discuss potential source(s) of inaccuracies in the results presented above.

### Incorrectly classified patterns

While the results presented in Tables 1 and 2 show outstanding performance by CNN (e.g., test accuracy of ~93% with *N* = 12000 for CNN4), the cluster indices of a few hundred testing samples (out of the 4000) have been incorrectly identified. From visually comparing examples of correctly and incorrectly identified patterns, inspecting the cluster centers in Fig. 1, or examining the results of Tables 1 and 2, it is not easy to understand why patterns from some clusters have been more (or less) frequently mis-classified. For example, in summer months using CNN4, patterns in cluster C2 (C4) are the most (least) frequently mis-classified. Patterns in C2 are most frequently mis-identified to belong to C3 (48 samples) while patterns in C3 are rarely mis-identified to belong to C2 (8). There are many examples of such asymmetries in mis-classification in Tables 1 and 2, although there are some symmetric examples too, most notably no mis-classification between C1 and C4 in summer or C2 and C3 in winter. It should be also noted that while CNN4 consistently has better overall test accuracy compared to CNN2 for summer/winter or as *N* changes, it may not improve the accuracy for every cluster (e.g. 915 C2 samples are correctly identified by CNN4 for summer months compared to 959 by CNN2). Visual inspection of cluster centers does not provide many clues on which clusters might be harder to re-identify or mix up; e.g., patterns in C2 in winter months are frequently (71 samples) mis-classified as C1 while rarely mis-classified as C3 (0) or C4 (9 samples) even though the cluster center of C2, which has a notable ridge over the eastern Pacific ocean and a low-pressure pattern over north-eastern Canada, is (visually) distinct from the cluster center of C1 or C3 but resembles that of C4.

While understanding *how* a CNN learns or *why* some patterns are identified and some are mis-identified can be of great interest for many applications, particularly those involving addressing a scientific problem, answering such questions is not straightforward with the current understanding of deep learning^59^. In the results presented here, there are two potential sources of inaccuracy: imperfect learning and improperly labeled patterns. The former can be a result of suboptimal choices of the hyperparameters or insufficient number of training samples. As discussed in Data and Methods, we have explored a range of hyperparameters manually. Still there might be room for further systematic optimization and improvement of the test accuracy. The results of Fig. 4 suggest that increasing *N* would have a small effect on the test accuracy. Training CNN4 for summer with *N* = 18000 increases the best test accuracy from 93.3% (obtained with *N* = 12000) to just 94.1%. These results suggest that the accuracy might be still further improved, though very slowly, by increasing *N*.

Another source of inaccuracy might be related to how the patterns are labeled using the K-means cluster indices. The K-means algorithm is deterministic and assigns each pattern to one and only one cluster index. In data that have well-defined classes, the patterns in each cluster are very similar to each other (high cohesion) and dissimilar from patterns in other clusters (well separated). However, in chaotic, correlated, spatio-temporal data, such as those studied here, some patterns might have similarities to more than one cluster, but the K-means algorithm assigns them to just one (the closest) cluster. As a result, two patterns that are very similar might end up in two different clusters and thus be assigned different labels. The presence of such borderline cases in the TR can degrade the learning process for CNN and their presence in the TS can reduce the test accuracy. The silhouette value *s* is a measure often used to quantify how a pattern is similar to its own cluster and separated from the patterns in other clusters^60^. Large, positive values of *s* indicate high cohesion and strong separation, while small and in particular negative values indicate the opposite.

To examine whether part of the inaccuracy in the testing phase is because of borderline cases, we show the percentage of samples correctly classified or incorrectly classified for a range of high and negative silhouette values in Table 3. The results indicate that poorly clustered (i.e. borderline) patterns, e.g. those with *s* < 0, are more frequently mis-classified compared to well-clustered patterns, e.g., those with *s* > 0.4 (11% versus 4.8%). This analysis suggests that part of the 6.7% testing error of CNN4 for summer months might be attributed to poor clustering and improper labeling (one could remove samples with low *s* from the TR and TS, but here we chose not to in order to have a more challenging task for the CNNs).

**Table 3:** Percentage of samples correctly classified or incorrectly classified for different ranges of silhouette values, *s*. Silhouette values, by definition, are between −1 and 1 and high (low and particularly negative) values indicate high (low) cohesion and strong (weak) separation. Percentages show the fraction of patterns in a given range of silhouette values. The samples are from summer months and for CNN4.

Silhouette value	*s* < 0	*s* > 0.2	*s* > 0.4
Correctly identified	89.0%	94.2%	95.2%
Incorrectly identified	11.0%	5.8%	4.8%

Note that soft clustering methods (e.g. fuzzy c-means clustering^61^) in which a pattern can be assigned to more than one cluster might be used to overcome the aforementioned problem if it becomes a significant source of inaccuracy. In any case, one has to ensure that the labels obtained from the unsupervised clustering technique form a learnable set for the CNN and be aware of the potential inaccuracies arising from poor labeling alone.

Finally, we highlight that we use the full Z500 fields, which as discussed earlier, contain non-stationary components. One may find more identifiable/predictable anomalies by removing such non-stationarity components, e.g., by removing the annul cycle. Here, we aim to assess the performance of CNNs in the presence of such non-stationarities.

### Performance of CNN and logistic regression for prediction

So far we have shown the performance of CNNs for re-identifying clustered weather patterns, which as discussed earlier, can be very useful for accelerated evaluation of different architectures and scaling of accuracy with the size of the training test. Here, we show the performance of CNNs for a problem that can be of practical importance: predicting the evolution of spatio-temporal climate/environmental patterns in the context of the cluster indices. Such cluster-based data-driven forecasting using machine learning methods or other techniques has been of rising interest in recent years^8,12,20,41^. Clustered precipitation or surface temperature patterns provide geographically cohesive regions of interest while clustered Z500 patterns often have connections with modes of climate variability. Therefore, predicting in terms of such clusters can be valuable. We emphasize that in the results shown below, CNNs are *not* used as a clustering technique, as clusters are already found using an unsupervised method (the K-means algorithm). Rather, CNNs are used to *predict* which cluster index a Z500 pattern will belong to in 1–5 days in the future.

We compare the performance of CNN4 with that of a simple machine learning method, logistic regression (Log-Reg), that has been used in some other studies for such cluster-based data-driven forecasting (see Herman *et al*.^12^ and references therein). The same training/testing procedure has been used for both methods. During training, the full Z500 patterns have been labeled based on the cluster index 1 day, 2 days ⋯ or 5 days later. During testing, a full Z500 pattern is inputted into the algorithm and the index of the cluster it would evolve to in 1 day, 2 days ⋯ is predicted. As shown in Table 4, CNN4 has the total prediction accuracy of 92.1% ± 0.4 (for lead day 1) to 76.4% ± 0.4 (for lead day 5) in summer and 93.3% ± 0.2 (for lead day 1) to 80.3% ± 0.2 (for lead day 5) in winter. CNN4 substantially outperforms Log-Reg, which has the total prediction accuracy of 65.3% ± 0.2 (for lead day 1) to 55.4% ± 0.2 (for lead day 5) in summer and 66.7% ± 0.3 (for lead day 1) to 61.7% ± 0.3 (for lead day 5) in winter. Table 5

**Table 4:** The overall test accuracy of predicting the cluster indices using CNN4 compared against the total accuracy using regular logistic regression algorithm (Log-Reg).

Lead days	CNN4 Summer	CNN4 Winter	Log-Reg Summer	Log-Reg Winter
Lead day 1	92.1% ± 0.4%	93.3% ± 0.2%	65.3% ± 0.2%	66.7% ± 0.3%
Lead day 2	89.3% ± 0.6%	91.1% ± 0.3%	63.2% ± 0.4%	65.8% ± 0.7%
Lead day 3	83.4% ± 0.2%	87.2% ± 0.2%	59.7% ± 0.2%	63.1% ± 0.3%
Lead day 4	80.1% ± 0.3%	82.4% ± 0.1%	56.8% ± 0.7%	63.2% ± 0.4%
Lead day 5	76.4% ± 0.4%	80.3% ± 0.2%	55.4% ± 0.2%	61.7% ± 0.3%

**Table 5:** Scaling of the overall test accuracy for prediction at lead day 1, lead day 3, and lead day 5 with sample size (*N*) for each of the methods (CNN4 and Log-Reg) for summer and winter.

Lead days	Sample Size	CNN4 Summer	CNN4 Winter	Log-Reg Summer	Log-Reg Winter
Lead day 1	*N* = 12000	92.1% ± 0.4%	93.3% ± 0.2%	65.3% ± 0.2%	66.7% ± 0.3%
	*N* = 8000	88.3% ± 0.4%	90.4% ± 0.4%	60.3% ± 0.3%	62.7% ± 0.5%
	*N* = 4000	87.6% ± 0.4%	86.5% ± 0.2%	52.3% ± 0.4%	54.6% ± 0.7%
Lead day 3	*N* = 12000	83.4% ± 0.2%	87.2% ± 0.2%	59.7% ± 0.2%	63.1% ± 0.3%
	*N* = 8000	81.3% ± 0.3%	86.4% ± 0.0%	52.3% ± 0.3%	57.7% ± 0.5%
	*N* = 4000	78.6% ± 0.5%	83.4% ± 0.3%	48.3% ± 0.5%	50.6% ± 0.4%
Lead day 5	*N* = 12000	76.4% ± 0.4%	80.3% ± 0.2%	55.4% ± 0.2%	61.7% ± 0.3%
	*N* = 8000	74.2% ± 0.2%	78.6% ± 0.5%	51.3% ± 0.3%	55.7% ± 0.5%
	*N* = 4000	71.1% ± 0.5%	74.4% ± 0.3%	45.3% ± 0.3%	49.6% ± 0.2%

shows the scaling of the prediction accuracy with the number of training samples (*N*) for both methods. As *N* is reduced by a factor of 3 from 12000 to 4000 for lead days 1–5, in summer, the accuracy of CNN4 declines 4.5–5.3% while the accuracy of Log-Reg declines 10.1–13%. Similarly, in winter, the accuracy of CNN4 (Log-Reg) declines 3.8–6.8% (12.1–12.5%). The results in Tables 4 and 5 show that CNN4 is superior, both in accuracy and scaling, to Log-Reg. Note that the accuracy of CNN4 for lead day 5 is above 70% for *N* = 4000, which is comparable to the number of training samples available for each season from high-quality reanalysis data since the beginning of the the satellite era.

The sources of inaccuracies for re-identification also contribute to in the inaccuracies in prediction. Furthermore, as expected, the prediction accuracy decreases with lead day. Note that here we attempt to predict Z500 only from knowing the earlier Z500 pattern. Including more variables, e.g., geopotential heights at other pressure levels, sea surface temperature, etc., might improve the prediction accuracy, especially at longer leads (see the discussion in Chattopadhyay *et al*.^20^). We leave this to future work.

## Discussion

In this paper, we first introduce an unsupervised auto-labeling strategy that can facilitate exploring the capabilities of supervised deep learning techniques such as CNNs in studying problems in climate and environmental sciences. The method can be applied to other deep learning pattern-recognition methods such as capsule neural networks^62^, and to any spatio-temporal data. The method enables one to examine the power and limitations of different architectures and scaling of their performance with the size of the training dataset for each type of data before further investing in labeling the patterns to address specific scientific problems, e.g. to study patterns that cause heat waves or extreme precipitation.

Second, we applied this strategy to clustered daily large-scale weather patterns over North America. We show the outstanding performance of CNNs in re-identifying and predicting patterns in chaotic, multi-scale, non-stationary, spatio-temporal data with minimal pre-processing. Building on the promising results of previous studies^46,47^, our analysis goes beyond their binary classifications and shows over 90% test accuracy for 4-cluster classification and prediction once there are at least 2000 training samples per cluster. The CNN is also shown to predict the evolution of Z500 patterns, in terms of cluster indices, with accuracy that is consistently higher, by around 25%, than that of a simpler machine learning technique, logistic regression. The auto-labeling strategy is used to examine how the re-identification or prediction accuracy scales with the number of training samples. This is an important question for practical purposes, as the perception that one needs large amount of data sometimes discourages using deep learning techniques. The scaling that is found here shows a nonlinear relation between accuracy and *N*, and suggests that the amount of data currently available from reanalysis since 1979 can be enough to successfully train an accurate CNN for applications involving daily large-scale weather patterns. While the scaling plots found here are very likely specific to this CNN architecture and dataset, the auto-labeling strategy enables one to easily generate such plots for their CNN and dataset before proceeding with a specific application.

The promising capabilities of CNNs in re-identifying and predicting complex patterns in non-stationary data with minimal pre-processing, and the potential for training of reanalysis data, can open frontiers for various applications in climate and environmental sciences. For example, the cluster-based forecasting of extreme events, which has been tried in some recent studies^8,12,20,41^, especially if conducted using a CNN trained on reanalysis data rather than model data, might lead to improved extreme weather prediction. The cluster-based forecasting of circulation patterns that is presented here, again if performed using a CNN trained on reanalysis data and using more input variables, might help with prediction of low-frequency variability in the subseasonal-to-seasonal timescales. As another example, CNNs, and methods involving feature extraction through subsequent layers of convolutions and pooling allow deep learning algorithms to extract patterns in the circulation that may otherwise be difficult to capture with traditional algorithms.Techniques such as recurrent neural networks (RNNs) with long short-term memory (LSTM) and tensor-train RNNs have shown encouraging skills in predicting time series in chaotic systems^42,63,64^. Coupling CNNs with these techniques can potentially provide powerful tools for spatio-temporal prediction; e.g., a convolutional LSTM network has been recently implemented for precipitation nowcasting^65^.

## Data and Methods

### Data from the large ensemble (LENS) community project

We use data from the publicly available Large Ensemble (LENS) Community Project^55^, which consists of a 40-member ensemble of fully-coupled atmosphere-ocean-land-ice Community Earth System Model version 1 (CESM1) simulations at the horizontal resolution of ~1°. The same historical radiative forcing from 1920 to 2005 is used for each member; however, small, random perturbations are added to the initial state of each member to create an ensemble. We focus on daily averaged geopotential height at 500 hPa (Z500). Z500 isolines are approximately the streamlines of the large-scale circulation at mid-troposphere and are often used to represent weather patterns^56^. We focus on Z500 from 1980 to 2005 for the summer months of June-August (92 days per summer) for all 40 ensemble members (total of 95680 days) over North America, 30°–90° north and 200°–315° east (resulting in 66 × 97 latitude–longitude grid points). Similarly, for winter we use the same 26 years of data for the months of December, January, and February (90 days per winter and a total of 95508 days).

### Clustering of weather patterns

The daily Z500 patterns over North America are clustered for each season into *n* = 4 classes. Following Vigaud *et al*.^9^, first, an EOF analysis is performed on the data matrix of zonal-mean-removed Z500 anomalies and the first 22 principal components (PCs), which explain 95% of the variance, are kept for clustering analysis. The K-means algorithm^54^ is used on these 22 PCs and repeated 1000 times with new initial cluster centroid positions and a cluster index *k* = 1, 2, 3 or 4 is assigned to each daily pattern.

It should be noted that the number of clusters *n* = 4 is not chosen as an optimal number, which might not even exist for these complex, chaotic, spatio-temporal data^17^. Instead, for the purpose of the analysis here, the chosen *n* should be large enough such that the cluster centers are reasonably distinct and there are several clusters to re-identify in order to evaluate the CNNs in a challenging multi-class classification problem, yet small enough such that there are enough samples per cluster for training and testing.

### Labeling and up/down-samplings

Once the cluster index for each daily pattern is computed, the full Z500 daily patterns are labeled using these indices for day 0, day − 1, ⋯ day − 5. We focus on the full Z500 fields, rather than the anomalies, for several reasons: (1) The differences between the patterns from different clusters are more subtle in the full Z500 compared to the anomalous Z500 fields; (2) The full Z500 fields contain all the complex, temporal variabilities and non-stationarity resulting from ocean-atmosphere coupling and changes in the radiative forcing while some of these variabilities might be removed by computing the anomalies; (3) One hopes to use CNNs with no or minimal pre-processing of the data. As a result of (1) and (2), re-identifying and predicting the cluster indices in the full Z500 fields provides a more challenging test for CNNs. As a result of (3), we focus on the direct output of the climate model, i.e., full Z500 field, rather than the pre-processed anomalies.

In our algorithm, the only pre-processing conducted on the data is the up-sampling/down-sampling shown in Fig. 3. The down-sampling step is needed to remove the small-scale, transient features of the chaotic, multi-scale atmospheric circulation from the learning/testing process. Inspecting the cluster centers in Fig. 1 shows that the main differences between the four clusters are in large scale. If the small-scale features, which are associated with processes such as baroclinic instability, are not removed via down-sampling, the CNN will try to learn the distinction between these features in different classes, which is futile as these features are mostly random. We have found in our analysis that without the down-sampling step, we could not train the CNN using a simple random normal initialization of the kernel weights (if instead of random initialization, a selective initialization method such as Xavier^66^ is used, the network can be trained for the full-sized images although the test accuracy remains low due to overfitting on small-scale features.) The need for down-sampling in applications of CNNs to multi-scale patterns has been reported previously in other areas^67^. In the applications that involve the opposite case, i.e. when the small-scale features are of interest and have to be learned, techniques such as localization can be used^68^.

Note that although Z500 is a scalar field, here we have used the three channels of RGB to represent it because we are focusing on only one variable. In the future applications, when several variables are studied together, each channel can be used to represent one scalar field, e.g. temperature and/or components of the velocity vector.

### Convolutional neural network (CNN)

The CNN is developed using the Tensorflow library^69^ following the Alex Net architecture^49^. We have trained and tested two CNNs: one with two convolutional layers, named CNN2, and a deeper one with 4 layers, called CNN4.

#### CNN2

The shallow neural network has two convolutional layers with 16 and 32 filters, respectively. Each filter has a kernel size of 5 × 5. In each convolutional layer, zero padding around the borders of images is used to maintain the size before and after applying the filters. Each convolutional layer is followed with a ReLU activation function and a max-pooling layer that has a kernel size of 2 × 2 and stride of 1 (stride is the number of pixels the filter shifts over in the pooling layer^45^). The output feature map is 7 × 7 × 64 which is fed into a fully connected neural network with 1024 neurons. The cross entropy cost function is accompanied by a *L*_2_ regularization term with a hyperparameter *λ*. Furthermore, to prevent overfitting, dropout regularization with hyperparameter *p* has been used in the fully connected layer. An adaptive learning rate *α*, a hyperparameter, is implemented through the ADAM optimizer^70^. The final output is the probability of the input pattern belonging to each cluster. A softmax layer assigns the pattern to the cluster index with the highest probability.

#### CNN4

The deeper neural network, CNN4, is the same as CNN2, except that there are four convolutional layers, which have 8,16,32 and 64 filters, respectively (Fig. 2). Only the last two convolutional layers are followed by max-pooling layers.

#### Training, validating, and testing procedures

For the case with *N* = 12000, 3000 labeled images from each of the four clusters are selected randomly (the TR set). Separately, 4 validation datasets, each with 1000 samples per cluster, are randomly selected. For the testing set (TS), 5 datasets, each with 1000 samples per cluster, are randomly selected. The TR, validation sets, and TS have no overlap. The equal number of samples from each cluster prevents class imbalance in training and testing.

**Figure 5:**
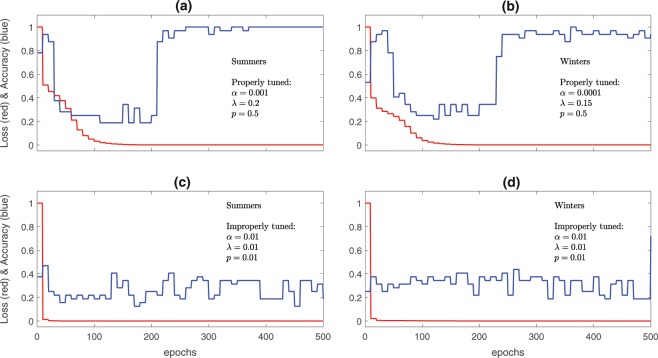
Examples of how loss and accuracy change with epochs during training for CNN4 for properly tuned and improperly tuned CNNs. Loss is measured as cross entropy normalized by its maximum value while the training accuracy is measured by the number of training samples correctly identified at the end of each epoch. Hyperparameters *α*, *λ*, and *p* are, respectively, the initial learning rate, regularization constant, and dropout probability. (**a**) *α* = 0.001, *λ* = 0.2 and *p* = 0.5 for summers with the test accuracy of 93.3%. (**b**) *α* = 0.001, *λ* = 0.15 and *p* = 0.5 for winters with the test accuracy of 93.8%. (**c**) *α* = 0.01, *λ* = 0.01 and *p* = 0.01 for summers with the test accuracy of 25%. (**d**) *α* = 0.01, *λ* = 0.01 and *p* = 0.01 for winters with the test accuracy of 60%). Several kernel sizes were tried and it was found that 5 × 5 kernel size gives the best validation accuracy and consequently the best test accuracy.

In the training phase, the images and their labels, in randomly shuffled batches of size 32, are inputted into the CNN and hyperparameters *α*, *λ*, and *p* are varied until small loss and high accuracy are achieved. Figure 5 shows examples of how loss and accuracy vary with epochs for properly and improperly tuned CNNs. Note that only an initial value of *α* is specified, which is then optimized using the ADAM algorithm. Once the CNN is properly tuned, the 4 validation sets are used to check the accuracy of CNN in re-identifying the cluster indices. If the accuracy is not high, *λ* and *p* are varied manually and training/validation is repeated until they both have similarly high accuracy. We found the best test accuracy with the hyperparameters shown in Fig. 5(a,b). Furthermore, we explored the effect of other hyperparameters such as the number of convolutional layers (from 2 to 8) and the kernel sizes (in the range of 5 × 5 to 11 × 11) in the convolutional layers on the performance of CNN for this dataset. We found that a network with more than 4 convolutional layers overfits on 12000 samples thus producing test accuracy lower than what is reported for CNN4 in Tables 1 and 2. Again, the best test accuracy is found with the architecture shown in Fig. 2 and described above.

In the testing phase, the best trained CNN is applied on the 5 datasets of TS once. The mean and standard deviation of the computed accuracy among these 5 datasets are reported in Tables 1 and 2.

For the cases with *N* = 500 to 8000, conducted to study the effect of the size of the training set *N* on the performance of CNN, *N*∕4 labeled images from each of the four clusters are selected randomly and used to train the CNN while testing is done on *N*∕8 (to the nearest integer) images from each class.

### Logistic-regression (Log-Reg)

The logistic-regression algorithm has been implemented as a baseline method to compare the performance of CNN4 following Herman *et al*.^12^ (where it has shown promising results). Logistic regression is essentially a one-neuron neural network with a softmax function as its activation. In order to ensure that Log-Reg does not overfit, an *L*_2_ regularization has been added to the logistic loss function^71^. The optimization has been performed with the ADAM optimizer similar to CNN4. The logistic regression code, just like CNN4, has been implemented in Tensorflow^69^.

### Alternative approach: applying CNN on data matrix rather than images

While CNNs are often used on images, even in applications to climate data^46,47^, they can be directly applied to the data matrices as well. For example, we can get the same accuracy as the CNN applied on images with CNN applied on a data matrix of labeled patterns. In such a data matrix *X*, each column contains the full Z500 over 97 × 66 grid points for each day. The CNN is applied to *X*, although the best results are obtained with a CNN whose architecture is slightly different from the one applied to images. In this case, the four convolutional layers have 8, 8, 16 and 32 filters while the fully connected layer has 200 neurons.

### Alternative approach: using EOFs or EOF-reduced data for training/testing

Given that we are interested in identifying or predicting the evolution of the large-scale patterns, one might attempt to first find a reduced feature space and then apply CNN or Log-Reg. EOF analysis is commonly used for dimension reduction of climate data. Here, we have conducted extensive experiments in which instead of training/testing on the full fields of Z500 we have:

1. Trained and tested CNN4 or Log-Reg on a number of leading EOFs of the Z500 data. For example, we have used the first 22 EOFs, which together explain 95% of the variance. We have tried using the leading EOFs that explain between 85% and 99% of the variance.
2. Trained and tested CNN4 or Log-Reg on patterns obtained from projecting (i.e., re-constructing) the Z500 pattern on a number of leading EOFs. Again, we have tried using the leading EOFs that explain between 85% and 99% of the variance.

Both approaches result in consistently lower accuracies (e.g., in Table 4, by as much as 25% for CNN4 and 10% for Log-Reg). We suspect that the loss of accuracy is due to the well-known shortcoming^30,72,73^ of EOFs, which are orthonormal by design, when applied to non-normal systems (in which dynamical modes are not normal to each other). Midlatitude circulation and many other geophysical flows are known to be non-normal^74–76^.

## Data Availability

The LENS dataset is publicly available at http://www.cesm.ucar.edu/projects/community-projects/LENS/.
